# Estimating Survival Functions in Children With Malaria in South Sudan: A Comparative Analysis of Associated Factors

**DOI:** 10.7759/cureus.79074

**Published:** 2025-02-15

**Authors:** Loro Gore Lado Jumi, Altaiyb Omer Ahmed Mohmmed

**Affiliations:** 1 Department of Statistics and Demography, School of Social and Economic Studies, University of Juba, Juba, SSD; 2 Department of Statistics, College of Science, Sudan University of Science and Technology, Khartoum, SDN

**Keywords:** kaplan-meier estimate, log-rank test, malaria, survival analysis, survival function

## Abstract

Introduction: Malaria remains one of the major health problems worldwide. It is a leading cause of morbidity and mortality in South Sudan, especially in children. The aim of this study was to estimate the survival functions of children with malaria and to compare these functions with respect to the associated factors.

Methods: This was a retrospective cohort study that utilized data extracted from records of children aged 1 month to 15 years who were diagnosed with malaria and admitted to Al Sabah Children's Hospital, Juba, South Sudan from 1 January to 31 December 2021. Kaplan-Meier was employed to estimate the survival functions, and the log-rank test statistic was used to compare survival curves with respect to categories of specific covariates.

Results: Out of 6,410 children diagnosed with malaria who were included in this study, 3,595 (56.08%) were males and 2815 (43.92%) were females. A total of 303 (4.73%) died and 6,107 (95.27%) were censored. The median survival time was three days (IQR 2-4), and the mean age of the study cohort was 23.66 months (95% CI: 23.01 to 24.32). Comorbidity status among children indicated that 1,552 (24.01%) had comorbidities during admission. With respect to the treatment covariate, 3,920 (61.15%) were treated with artesunate. Kaplan-Meier survival curves showed increased survival for children treated with quinine and those without comorbidity. The log-rank test revealed that the covariates treatment and comorbidity were significant (p < 0.05), both having an impact on survival among children.

Conclusion: This study found that the median survival time for children with malaria was three days. Additionally, comorbidity decreased survival among children. Treatment with quinine showed better survival rates compared to artesunate, despite studies suggesting artesunate as a replacement for quinine. Strengthening the healthcare system, provision of good-quality drugs, and implementing control intervention measures to reduce the transmission of malaria are essential for improving child survival in the country. Furthermore, it is essential to carry out a comparative study of quinine and artesunate for the treatment of malaria. Future studies on malaria in South Sudan have to be conducted by using a prospective study design to address the challenges of incomplete data and potential biases.

## Introduction

Malaria is a common vector-borne parasitic infectious disease caused by five protozoan parasite species of the genus *Plasmodium*. It remains one of the major health problems worldwide, particularly in sub-Saharan Africa, despite the significant progress in reducing malaria incidence during the last two decades. Globally, there were an estimated 241 million malaria cases in 2020 in 85 malaria-endemic countries, increasing from 227 million in 2019. This represents an additional 14 million cases, with the majority of the increase coming from countries in the WHO African Region. These countries accounted for about 95% of cases and 96% of deaths worldwide. It is worth noting that 80% of all deaths in this region are among children aged under five years. The increase in cases in 2020 can be attributed to the disruption of healthcare services during the COVID-19 pandemic [1].

Despite control intervention measures to reduce transmission of malaria and its socio-economic impact globally, the sub-Saharan Africa region is facing challenges in reducing the intensity of the disease, especially in remote areas where there is a lack of understanding of the disease epidemiology, a weak health system, scarcity of control intervention measures, and high poverty levels, among other factors [2]. Long-lasting insecticide-treated nets (ITNs) and indoor residual spraying (IRS) are regarded as the key malaria prevention and control interventions because of their proven impact on the reduction of disease burden. Protecting high-risk groups, such as pregnant women and children, through antimalarials also reduces the disease burden in endemic countries [3].

Other issues regarding malaria prevention and control persist, including a growing rate of mosquitoes' resistance to the pyrethroids used in ITNs and parasites' resistance to antimalarials, resulting in difficulty in curbing the disease. Additionally, the health system is characterized by limited access to medical services and products, a lack of human resources for health, a shortage of functioning health facilities, and an overall lack of quality healthcare service delivery [4]. 

A retrospective study was conducted to determine the prevalence and outcome of malaria among hospitalized children in Al Sabah Children's Hospital, South Sudan. The study utilized data extracted from hospital records. The results indicate that the prevalence of malaria among hospitalized children was 78%. Severe malaria alone affected 28% of the children and accounted for 14% of the deaths. On the other hand, severe malaria in combination with other diseases affected 50% of the children and accounted for 58% of the children who died [5].

Malaria is endemic in South Sudan, with transmission occurring throughout the year. It is a major cause of morbidity and mortality, particularly in children. *Plasmodium falciparum* is the predominant causative organism. In 2021, South Sudan ranked among the top 22 countries with the highest burden of malaria in the world, accounting for 1.2% of all global malaria cases and deaths. In East and Southern Africa, 5% of malaria cases occurred in South Sudan [1]. The prevalence of malaria in children aged 6-59 months increased slightly from 30% in 2013 to 32% in 2017, according to the South Sudan Malaria Indicator Survey [6]. The disease accounts for about 66.8% of all health facility visits in outpatient departments, 30% of all hospital admissions, and 50% of all causes of death in hospitals [7]. Further research is needed to understand the factors influencing malaria. Therefore, this study aimed to estimate the survival functions of children with malaria and to compare these functions with respect to the associated factors. 

## Materials and methods

This was a retrospective cohort study that utilized data extracted from records of children aged 1 month to 15 years. The children included in the study were those diagnosed with malaria and admitted to Al Sabah Children's Hospital in Juba, South Sudan. The data collected were from 1 January to 31 December 2021. In this study, the response (or dependent) variable was the survival time of a child with malaria. The covariates included age, gender, body weight, season, place of residence, treatment, and comorbidity. Kaplan-Meier curves was employed to estimate the survival functions, and the log-rank test statistic was used to compare survival curves with respect to categories of specific covariates.

Description of variables

The continuous variables in this study include survival time, defined as as the time from admission to death of a child due to malaria (the event of interest) or censoring (in days); the age of child diagnosed with malaria (in months); body weight of a child (kilograms). The categorical variables include gender (coded as 1 for male and 0 for female), season (dry season from December to March and wet season from April to November, coded as 1 for wet and 0 for dry), treatment (coded as 1 for quinine and 0 for artesunate), residence (coded as 1 for urban and 0 for suburban), and comorbidity (coded as 1 if present and 0 if not). All data analyses were performed using STATA version 14.1 Special Edition.

Ethical considerations 

The ethical approval was obtained from the Ministry of Health, Research Ethics Review Board (MOH/RERB), Juba, Republic of South Sudan, approval no. (MOH/RERB 55/2022, dated 30/09/2022). The data collected were anonymized, and the patients’ confidentiality was maintained.

Survival analysis

Survival analysis is a collection of statistical procedures for data analysis of time until an event occurs. An event can refer to death, disease incidence, recovery, or any designated experience of interest that may happen to an individual [8]. One important feature of survival analysis is called censoring, which occurs when we do not know the time an event of interest occurs for an individual. The reasons for censored survival time data may include an individual not experiencing the event before the study ends, being lost to follow-up, or withdrawing from the study. Survival analysis depends on the distribution of survival time. Two fundamental functions that characterize the distribution are the survival function and the hazard function.

 Survival Function

The survival time denoted by t of an individual is an observed value of a random variable T, that can take nonnegative values. Supposing that T has probability distribution with probability density function f(t), the distribution function of T is defined by the survival time denoted by t of an individual is an observed value of a random variable T, that can take nonnegative values. Supposing that T has probability distribution with probability density function, the distribution function of T is defined as follows:

$F(t) = P(T &lt; t) = \int_{0}^{t} f(u) \, du \tag{1}$

which represents the probability that the survival time is less than some value "t". 

The survival function S(t) is the probability that an individual survives beyond specified time point t, that is, experiencing the event after time t. It is defined by

$S(t) = P(T > t) = 1 - F(t) \tag{2}$

Once the survival function has been estimated, an estimate of median survival time can be obtained. The time beyond which 50% of the individuals in a study population are expected to survive is given by the value $t\left( 50 \right)$, which is such that $S\left\{ t\left( 50 \right) \right\} = 0.5.$

Hazard Function

The hazard function h(t) is the probability that an individual experience an event in an interval after time t, given that the individual survived to that time. It is defined by

$h(t) = \lim_{\Delta t \to 0} \frac{P(t \leq T \leq t + \Delta t \mid T \geq t)}{\Delta t} \tag{3}$

The hazard function is sometimes called the hazard rate, the intensity function or the force of mortality [8,9].

Kaplan-Meier method

Kaplan-Meier (or product limit) estimator is a nonparametric method commonly used to estimate survival functions from censored and uncensored survival data. Suppose that there are n individuals with observed survival times $t_{j}, j=1,2, ... ,n$,  and that there are m event times where $m\le n$​​​ arranged in ascending order $t_{(1)}, t_{(2)}, ... ,t_{(m)}$​​​​​​. Let the number of individuals at risk of event just before time $t_{(j)}$​​​​ be denoted by $n_{j}$ and the number of observed events be denoted by $d_{j}$. Therefore, the Kaplan-Meier estimator of the survival function at time t is given by

$\hat{S}(t)=\prod_{t_{(j)}\le t}^{}(\frac{_{n_{j}-d_{j}}}{n_{j}}) \tag{4}$

Where $\hat{S}(t) = 1$ for $t &lt; t_{(1)}$ [9,10]. 

Log-rank test

The log-rank test also referred to as (Mantel-Cox) is a nonparametric method for comparing two or more survival curves. It is the most widely used method in survival analysis to test for significant difference between survival curves. The log-rank statistic is a large sample chi-square test that makes use of the observed and the expected number of events at time $t_{j}$. Thus, for each event time in the entire set of data, let;

$n_{ij}=$ the number of individuals in group (i)  who are at risk of experiencing the event at time $t_{(j)}$

$m_{ij}=$ the number of individuals in group (i) who experience the event at time $t_{(j)}$​​​​​​

Then the expected number of individuals in group (i) who experience the event at time $t_{(j)}$​​​​​ is given by

$e_{ij} = \left( \frac{n_{1j}}{n_{1j} + n_{2j}} \right)(m_{1j} + m_{2j}) \tag{5}$

The log-rank test is defined by 

Log-rank test $= \frac{(O_{i} - E_{i})^{2}}{\mathrm{Var}(O_{i} - E_{i})} \tag{6}$

$\mathrm{Var}(O_{i} - E_{i}) = \sum\nolimits_{j} \frac{n_{1j}n_{2j}(m_{1j} + m_{2j})(n_{1j} + n_{2j} - m_{1j} - m_{2j})}{(n_{1j} + n_{2j})^{2}(n_{1j} + n_{2j} - 1)} \tag{7}$

Where $O_{i}-E_{i}=\sum_{j=1}^{n}(m_{ij}-e_{ij}) ~~i=1,2$

 $O_{i}$ is the observed number of events in the group (i) and $E_{i}$ is the expected number of events in group (i)  [8].

## Results

Out of 6,410 children diagnosed with malaria who were included in this study, 3,595 (56.08%) were males, 303 (4.73%) died, and 6,107 (95.27%) were censored. The median survival time was three days (IQR 2-4) (Table 1), the mean age of the study cohort was 23.66 months (95% CI: 23.01 to 24.32), and the mean weight was 9.93 kg (95% CI: 9.80 to 10.06) (Table 2). Comorbidity status among children indicated that 1,552 (24.01%) had comorbidities during admission. With respect to the treatment covariate, 3,920 (61.15%) were treated with artesunate (Table 3). Percentages and frequencies were used to summarize categorical covariates. 

**Table 1:** Summary statistics of survival time of children diagnosed with malaria

Covariate	Median	Lower quartile	Upper quartile	Minimum	Maximum
Time	3	2	4	1	10

**Table 2:** Summary statistics of continuous covariates of children diagnosed with malaria

Covariate	Mean	Standard error	95% CI (lower, upper)	
Age (months)	23.66415	0.3357715	23.00593	24.32237
Weight (kg)	9.928958	0.0660853	9.799409	10.05851

**Table 3:** Distribution of categorical covariates of children diagnosed with malaria

Covariate	Frequency	Percentage
Gender		
Male	3595	56.08
Female	2815	43.92
Season		
Wet	4014	62.62
Dry	2396	37.38
Treatment		
Quinine	2490	38.85
Artesunate	3920	61.15
Place of residence		
Urban	3434	53.57
Suburban	2976	46.53
Comorbidity		
With comorbidity	1552	24.21
Without comorbidity	4858	75.79

**Figure 1:**
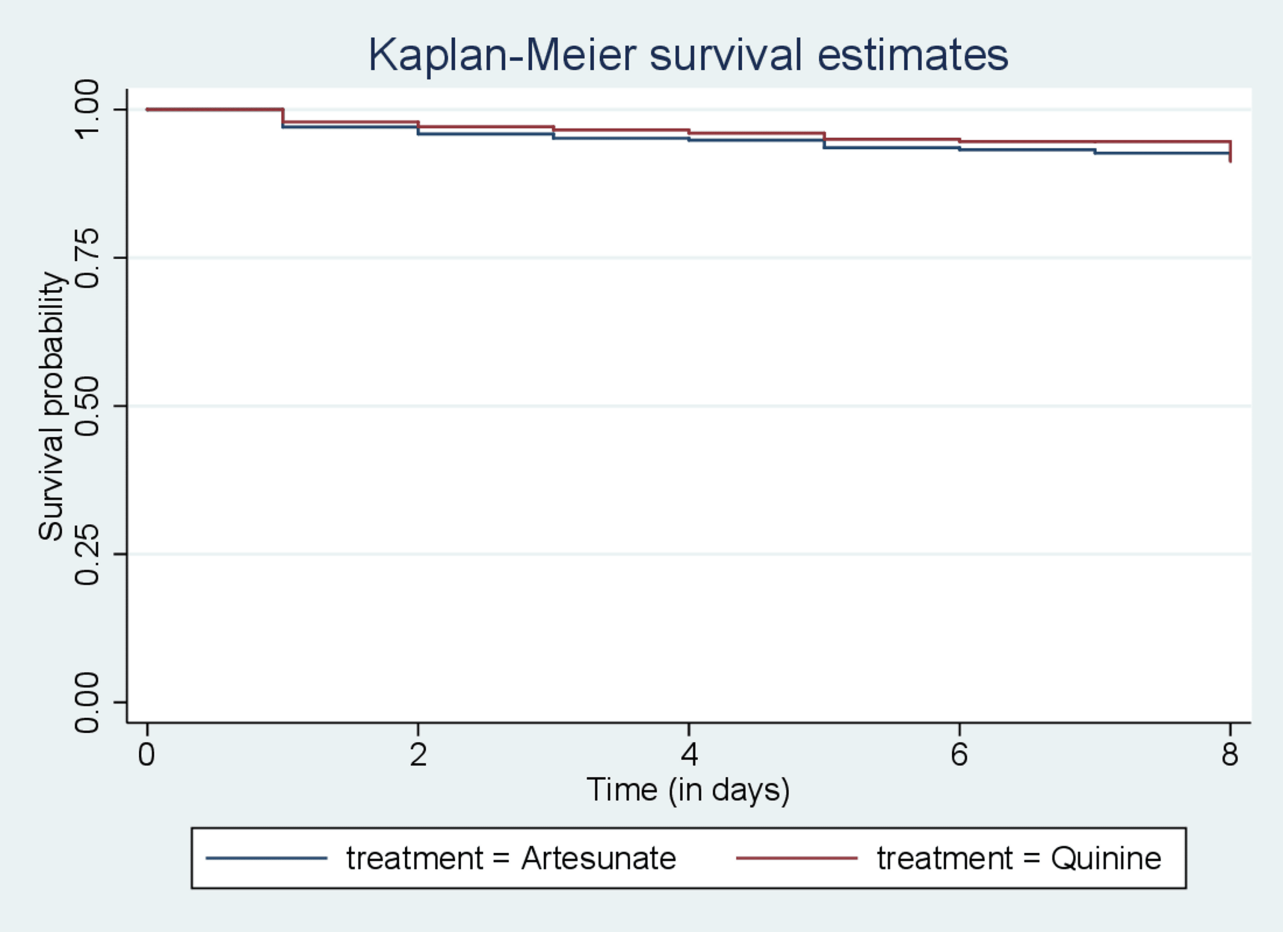
Kaplan-Meier curves of the children treated with artesunate and children treated with quinine

**Figure 2:**
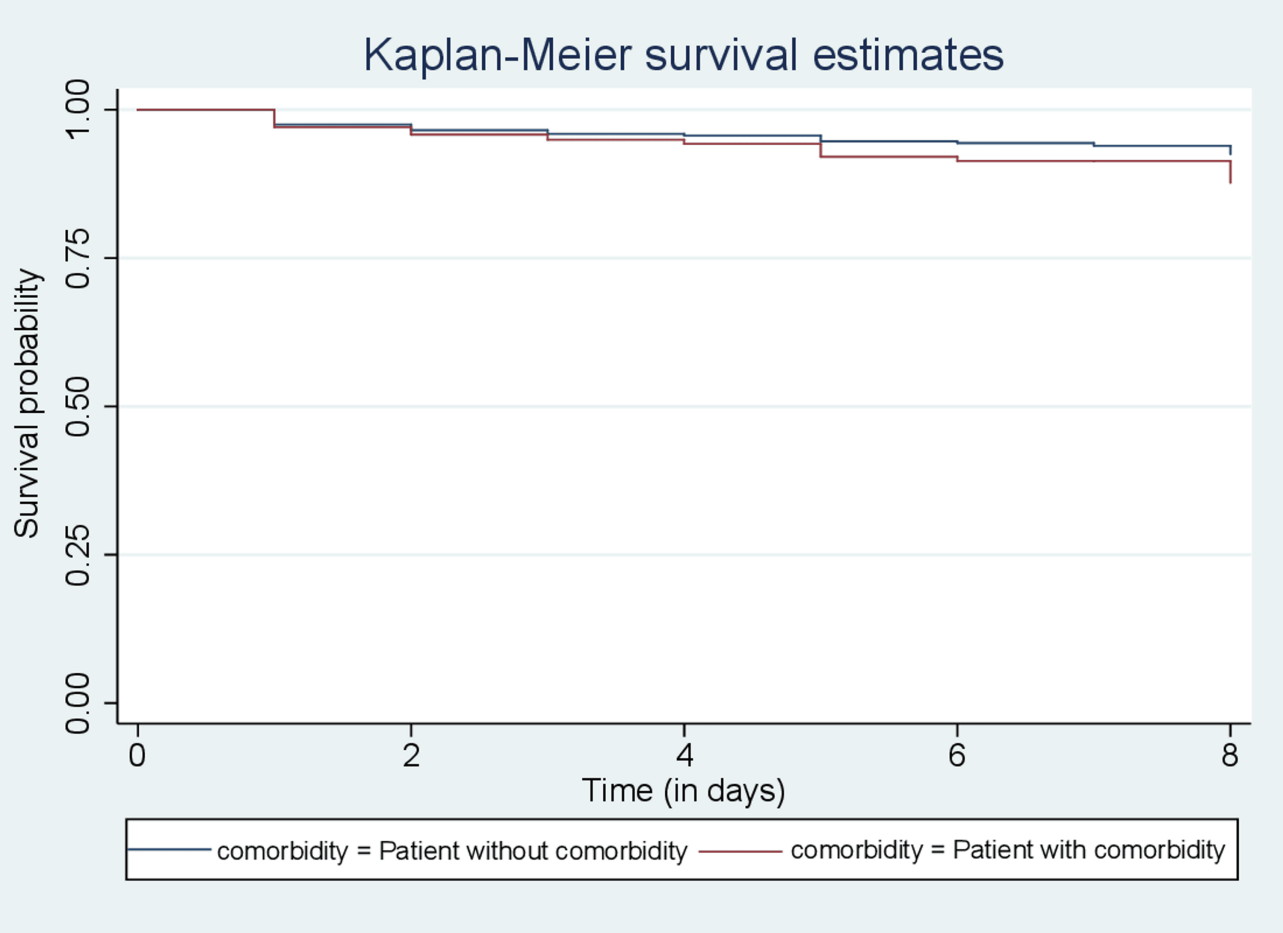
Kaplan-Meier curves of the children without comorbidity and children with comorbidity

The curves in Figure 1 show that the survival probabilities in the first days appeared to be the same but later the differences showed up clearly the curve of quinine consistently lies above that of artesunate. This reveals that children who were treated with quinine have better survival chances than those treated with artesunate. Also, in Figure 2, the curve for children without comorbidity consistently lies above that of those with comorbidity, indicating better survival chances for the former.

The log-rank test was used to determine the significant difference between survival functions of categories of specific covariates. Based on Table 1, it is clear that the log-rank test was not significant in the survival probability between the categories of the covariates gender (p-value = 0.1729), season (p-value = 0.5795) and place of residence (p-value = 0.1466) with (p > 0.05). This means these covariates do not have an influence on the survival probability of children with malaria. The test was significant (p < 0.05) in survival probability between the categories of the covariates, treatment and comorbidity. This means treatment and comorbidity have an influence on the survival time of children with malaria

**Table 4:** Results of the log-rank test for categorical covariates of children with malaria A p-value of less than 0.05 is considered significant.

Covariate	Observed events	Expected events	Chi-square value	P-value
Gender			1.86	0.1729
Male	145	133.34		
Female	158	169.66		
Season			0.31	0.5795
Wet	186	190.61		
Dry	117	112.39		
Treatment			5.62	0.0178
Quinine	94	113.71		
Artesunate	209	189.29		
Place of residence			2.11	0.1466
Urban	147	159.48		
Suburban	156	143.52		
Comorbidity			4.10	0.0428
With comorbidity	85	70.29		
Without comorbidity	218	232.71		

## Discussion

The aim of this study was to estimate the survival functions of children with malaria and to compare these functions with respect to the associated covariates. Also, to identify the covariates that have a significant impact on the survival time of children with malaria admitted to Al Sabah Children Hospital in Juba, South Sudan.

The study showed that the median survival time was three days (IQR 2-4). This result is consistent with the result of a study conducted in Germany on factors associated with hospital length of stay in patients with malaria by Hoffmeister [11]. However, a study by Ariyo et al. [12] on the application of survival analysis among children with malaria in Nigeria reported a median survival time of 11 days. This may be attributed to differences in the study settings, severity of malaria, sample size, standard of care provided, availability of medical resources, or treatment modality. Median survival time is reported in most studies that used survival analysis because of censored data, and the median is a better measure of centrality than the mean. Additionally, it is not possible to know when patients who are alive at the end of the study will have the event of interest, so the mean cannot be calculated [13].

The covariates gender, residence and season were found not to be significant with p-values (p > 0.05). This means that these covariates do not influence the survival time of children with malaria. The covariate comorbidity was found to significantly influence survival probability with a p-value (p < 0.05), which indicates that survival was lower among children with comorbidities than those without. This is similar to the results of studies conducted by Bekele et al. [14] among malaria patients admitted to Arba Minch General Hospital in Ethiopia and Waxman et al. [15] on characteristics and survival of patients with Ebola virus infection, malaria, or both in Sierra Leone.

This study revealed that treatment was a significant covariate for survival, with a p-value (p < 0.05). This suggests that children with malaria who were treated with quinine are found to have a higher survival probability than those treated with artesunate. This is consistent with the result of a study by Patel et al. [16] that compared the use of quinine versus artesunate for the treatment of severe falciparum malaria, concluding that quinine is preferable.

However, it is inconsistent with results obtained in other studies. Ngbonda et al. [17] studied artesunate versus quinine in the treatment of severe *Plasmodium falciparum* malaria in the northeastern region of the Democratic Republic of Congo, focusing on parasites and fever clearance. Their results showed that artesunate and quinine are comparable alternatives in the treatment of severe malaria, and concluded that artesunate and quinine present a similar clinical and biological efficiency. Eltahir et al. [18] compared artesunate and quinine in the treatment of Sudanese children with severe *Plasmodium falciparum* malaria. Results revealed that there was no significant difference in the fever, parasite clearance, coma resolution, as well as child survival. Additionally, it has been contrary to the results of studies by Dondorp et al. [19] that conducted an open-label, randomized trial to compare parenteral treatment with either artesunate or quinine in African children with severe malaria. They reported that artesunate substantially increased child survival compared to quinine, and Sagbo et al. [20] found that the treatment of severe malaria in children with artesunate is superior to quinine. This includes reductions in the duration of parenteral treatment and the risk of death in Benin [20].

This variation in the results is probably due to differences in practices, including not applying strict criteria for severe malaria, high malaria transmission settings, the endemic nature of the disease, quality of artesunate used, parasite drug resistance, and poor quality drugs which remain a problem worldwide. Another possible explanation for the artesunate treatment results is poor compliance.

The study has some limitations. As a single hospital-based study, the results may not be generalizable to the general population. The retrospective nature of the study depends on hospital records that may have incomplete information, which could influence the results.

## Conclusions

This study found that the median survival time was three days for children with malaria. The covariates of gender, residence, and season were found not to have a significant impact on the survival time of children with malaria. Additionally, it revealed increased survival among children without comorbidities compared to those with comorbidities. It also showed that children treated with quinine have better survival compared to artesunate, despite studies suggesting artesunate as a replacement for quinine. Quinine continues to play a role in the treatment and management of malaria in South Sudan. Strengthening the healthcare system, providing good quality drugs, and implementing control intervention measures to reduce the transmission of malaria are essential for improving child survival in the country. Furthermore, it is essential to carry out a comparative study of quinine and artesunate for the treatment of malaria. Future studies on malaria in South Sudan have to be conducted by using a prospective study design to address the challenges of incomplete data and potential biases.
